# Characterising Hydroxyapatite Deposited from Solution onto Novel Substrates: Growth Mechanism and Physical Properties

**DOI:** 10.3390/nano13172483

**Published:** 2023-09-03

**Authors:** Bríd Murphy, Jhonattan Baez, Mick A. Morris

**Affiliations:** 1 Advanced Materials & Bioengineering Research Centre (AMBER), Trinity College Dublin, Dublin 2, D02 CP49 Dublin, Ireland; baezj@tcd.ie; 2 School of Chemistry, Trinity College Dublin, Dublin 2, D02 PN40 Dublin, Ireland

**Keywords:** hydroxyapatite, solution deposition, orthopaedic coatings, novel substrates

## Abstract

Whilst titanium, stainless steel, and cobalt-chrome alloys are the most common materials for use in orthopaedic implant devices, there are significant advantages in moving to alternative non-metallic substrates. Substrates such as polymers may have advantageous mechanical biological properties whilst other substrates may bring unique capability. A key challenge in the use of non-metal products is producing substrates which can be modified to allow the formation of well-adhered hydroxyapatite films which promote osteointegration and have other beneficial properties. In this work, we aim to develop methodology for the growth of hydroxyapatite films on surfaces other than bulk metallic parts using a wet chemical coating process, and we provide a detailed characterisation of the coatings. In this study, hydroxyapatite is grown from saturated solutions onto thin titanium films and silicon substrates and compared to results from titanium alloy substrates. The coating process efficacy is shown to be dependent on substrate roughness, hydrophilicity, and activation. The mechanism of the hydroxyapatite growth is investigated in terms of initial attachment and morphological development using SEM and XPS analysis. XPS analysis reveals the exact chemical state of the hydroxyapatite compositional elements of Ca, P, and O. The characterisation of grown hydroxyapatite layers by XRD reveals that the hydroxyapatite forms from amorphous phases, displaying preferential crystal growth along the [002] direction, with TEM imagery confirming polycrystalline pockets amid an amorphous matrix. SEM-EDX and FTIR confirmed the presence of hydroxyapatite phases through elemental atomic weight percentages and bond assignment. All data are collated and reviewed for the different substrates. The results demonstrate that once hydroxyapatite seeds, it crystallises in the same manner as bulk titanium whether that be on a titanium or silicon substrate. These data suggest that a range of substrates may be coated using this facile hydroxyapatite deposition technique, just broadening the choice of substrate for a particular function.

## 1. Introduction

The most commonly used materials for knee or hip replacements are stainless steel, cobalt-chromium, or titanium alloys due to their mechanical strength and bio-inertness [1,2,3,4]. Although it is a common metal for use in orthopaedic implants, there are concerns surrounding titanium’s (and the other metals’) use. Specifically, the disadvantages of titanium parts include surface propagated cracks, titanium leeching, patient allergies, and mechanical requirements that require the addition of other metals [5,6,7]. There is also a problematic difference in the Young’s Modulus of the implant alloy and natural bone (somewhat worse for Co-Cr) because of a higher density of the metals [8,9,10,11,12].

Research over the last decade has focused on alternatives to titanium alloys. Dental applications often use zirconia-based implants and researchers are showing data whereby these could replace titanium for bulk orthopaedic implants since they have the same mechanical properties but improved antimicrobial and osteointegrative properties [13,14]. Polymers have considerable potential for use in orthopaedic implants due to their mechanical properties, density similarity to bone, and biocompatibility [15,16]. Experiments on epoxy-coated bamboo fibres found that it had potential as an implant material that would incur lower aseptic loosening post-joint replacement because its stiffness is close to that of natural bone [17]. Biodegradable magnesium-based alloys have considerable potential for orthopaedic implants but on the lower size scale, such as screws or clips, not large joints [18,19]. The advent of 3D printing has opened huge opportunities to produce patient-specific orthopaedic implants from metals or composite-type materials [20,21,22,23]. Despite numerous investigations of alternative, promising materials, many of these require very different processing methods and modification, including, most importantly, surface modification [24]. This is because a key barrier to the use of new substrate materials such as plastics for implant devices is surface functionality, which enables covalent bonding to bone. Specifically, the development of materials for orthopaedic parts is hindered by the challenges of developing osteoinductive hydroxyapatite (HA) layers at the substrate surface which is well-developed for metal parts, particularly titanium [25,26,27].

There are various long-standing methods of depositing HA onto metallic orthopaedic parts such as chemical vapour deposition, electro spraying, and plasma spraying [28,29,30,31]. These are often inconsistent with the use of alternative materials. The plasma spraying of HA, the established industry method, has been evaluated to coat novel materials such as polyetheretherketone (PEEK), bioresorbable Mg alloys, or zirconia implants [32,33,34]. A key parameter outlined for successful coating of Mg-based and composite materials with HA by plasma or cold-spraying is the surface hydrophilicity of the substrate [35,36,37]. In general for most polymer types of parts, the plasma spraying of hydroxyapatite is impeded by this disparity in hydrophilicity but also the temperatures required for plasma spraying are incompatible with polymers [38,39]. There are studies published whereby the plasma spraying of HA onto polymers with a varying degree of success is assisted by other materials such as nanoparticles or carbon-based materials [40,41,42,43]. Electrochemical methods of depositing HA exist but are an obvious challenge for non-conductors [44,45]. Laser deposition methods of depositing HA have shown potential for titanium alloys but are challenged by scale, the formation of intermediary layers between the part, and also substrate temperature issues for coating for non-metallic parts [46,47,48].

As outlined, there is a clear need to develop appropriate HA coating methods for non-metallic surfaces that result in coatings with similar properties to metallic coatings and can be readily scaled. Solution-based methods have been explored for HA powder formation but have no widespread application as a coating technique [49,50]. However, hydrothermal synthesis of the HA coating has shown promise on novel materials, such as magnesium, but requires high temperature and pressure [51,52,53]. Correspondingly, solution-based HA deposition, which uses a material containing polymeric particles, could be deployed as a gateway to implementing polymeric-based implants [54]. Other solution-based techniques rely on the use of simulated body fluids [55,56]. However, the composition and bioactivity of these simulated bodily fluids often vary across studies [57,58]. Under solution, the formation of HA occurs at active sites, whereby mineral precipitation begins [59]. To this end, we postulate that if we can generate active sites on novel substrates, using a colloidal-solution-based coating process, we can grow a coherent HA layer on these substrates. This new and exciting solution-based coating technique has never been studied as a direct solution for substrate technique. The main objective of this work is to apply this novel coating technique to different substrates and to rationalise the HA film in terms of growth mechanisms and chemical composition. The aim is to show the efficacy of this HA coating system for non-bulk Ti and highlight this system as having great potential for change in the material of orthopaedic implants.

The research presented in this paper demonstrates a facile and scalable solution for the deposition of HA onto non-metallic substrates that would enable the effective coating of non-traditional orthopaedic substrates. Unlike other studies, this work probes the adhesion of HA films as they evolve on novel substrates without any significant pre-preparation or the use of additional materials. The crystal phase, porosity, and integrity of the coating are compared to existing HA thin films (on titanium). Titanium coupons (Ti coupons) are used to model standard orthopaedic parts. Novel substrates (planar silicon and titanium thin films) were used as indicators that the methodology could form HA films formed on non-metals. All substrates that undergo basic activation are studied afterwards physically to assess their roughness and water contact angles, but also chemically to determine if the pre-activation has caused any intermediate layers. We present the results of an extensive XPS study that shows how varying the surface activation of Ti thin-film substrates gives rise to HA elements such as Ca, P, and O attaching to the surface in difference chemical bond states. Electron microscopy data compare the films of HA which are formed on the three different substates from this solution process. The crystallinity of the HA formed is assessed at different stages in film growth, and from it a clear picture emerges of the phase evolution of the HA from this process. It is clear that HA grows in the same manner for titanium bulk parts and Ti thin-film parts, which have characteristic HA lattice peaks and a dominant calcium-deficient peak. However, the silicon part’s growth is less characteristic than HA mineral growth. Further transmission election data show that the thickness of the Ti-formed HA films are comparable and that all substrates have HA mineral material with polycrystalline areas within an amorphous matrix.

The results suggest that these methods should be further explored as a way to develop functional alternative implant devices. The data prove that a <100 nm titanium layer, which could be applied to any material, will allow for HA film growth. The HA film will be fully aligned with the HA growth on a bulk titanium part when is it deposited using this colloidal solution system. Separately, while the silicon parts do not give rise to HA films as heterogeneously as the Ti parts, we have shown that once the HA seeds, it evolves in a similar manner to HA on Ti. This finding proves that this coating method is easily applied to other materials once an activation is applied. 

## 2. Materials and Methods

All materials and reagents were used as received. Monobasic potassium phosphate (KH_2_PO_4_) United States pharmacopeia (USP) reference standard, Honeywell Fluka hydrochloric (HCl) acid solution 6 M, tris(hydroxymethyl)-aminomethane (TRIS) ACS reagent, 99.8% sodium chloride (NaCl) BioXtra, and 99.5% calcium nitrate tetrahydrate (Ca(NO_3_)_2_·4H_2_O) ACS reagent were all obtained from Sigma Aldrich. KH_2_PO_4_, TRIS, and NaCl were mixed in deionised water (DIW) to yield a supersaturated phosphate solution. HCl was added to increase solubility, stability, and prevent precipitation. Ca(NO_3_)_2_·4H_2_O was mixed with DIW to yield a supersaturated calcium solution. For deposition, the supersaturated solutions were combined before dilution by a factor of 10–20 and with warming to 40–50 °C. The mixture was then agitated in a reaction vessel. Four-inch silicon wafers were used as received and a subset of these underwent e-beam evaporation–deposition (Temescal FC-2000) to generate 100 nm thin titanium films. Titanium coupons of a Ti-6Al-4V alloy were also used. All substrates were submerged in hot basic solutions to increase their roughness and foster a more negatively charged surface to which calcium ions could attach [60,61]. Substrates were placed in the reaction vessel for deposition and then removed and rinsed with DIW. This process was repeated several times with fresh solutions to grow a coherent layer of HA at the solution–substrate interface. For characterisation, some samples are analysed after one solution deposition run and some are analysed after up to six solution deposition runs. 

Scanning electron microscopy (SEM) data were collected using a Carl Zeiss Ultra Microscope equipped with an in-lens detector. An accelerating voltage of 5 to 10 kV was used. Energy-dispersive X-ray spectroscopy (EDX) spectra were acquired at 15 kV on an Oxford Inca EDX detector. Water contact angles (WCAs) of the samples were measured on a custom-built device using a fast shutter camera and a 60 Hz sampling rate. Ten microlitre drops of pure water were placed on the sample. Image J software (version 1.4.3) with DropSnake plug-in was used to measure the water contact angle. Atomic force microscopy (AFM) was performed using an aXE-7, Park Systems AFM with non-contact cantilevers. AFM images we imported to Parks Systems’ XEI imaging software (version 4.3.0 Build 5) which allows for quantitative and statistical analyses of images. Using the ‘Region’ tab, we calculated average roughness values as average roughness (Ra) and peak-to-valley roughness (Rpv) for each substrate from their corresponding AFM images. X-ray Photoelectron Spectroscopy (XPS) data were gathered using an Al Kα X-ray source, 1486.6 eV, CTX400 (PSP Vacuum Technology) with ultra-high vacuum conditions (<5 × 10^−10^ mbar). Each spectrum was calibrated using a C 1s binding energy of 284.8 eV. Analysis was carried out using CasaXPS software (version 2.3.23.PR1.0). X-ray diffraction (XRD) patterns were acquired using a Bruker Advance Powder Diffractometer (Cu-Kα radiation with λ = 1.5406 Å, operating voltage of 40 kV, and current of 40 mA). Measurements were performed in the 2θ range from 10° to 60° at steps of 0.004°. Transmission electron microscopy (TEM) was performed on an FEI Titan 80–300 microscope. Lamellae for TEM cross-section images were prepared using a Zeiss AURIGA Focused Ion Beam (FIB), obtaining accelerating voltages of 5–30 kV and ion beam currents of 50 pA–2 nA. Fourier-transform infrared spectroscopy (FTIR) spectra were obtained using a Perkin Elmer’s Spotlight 200i benchtop device with attenuated total reflectance (ATR, 4000–500 cm^−1^, 8 scans, and 4 cm^−1^ resolution diamond crystal).

## 3. Results

**Figure 1:**
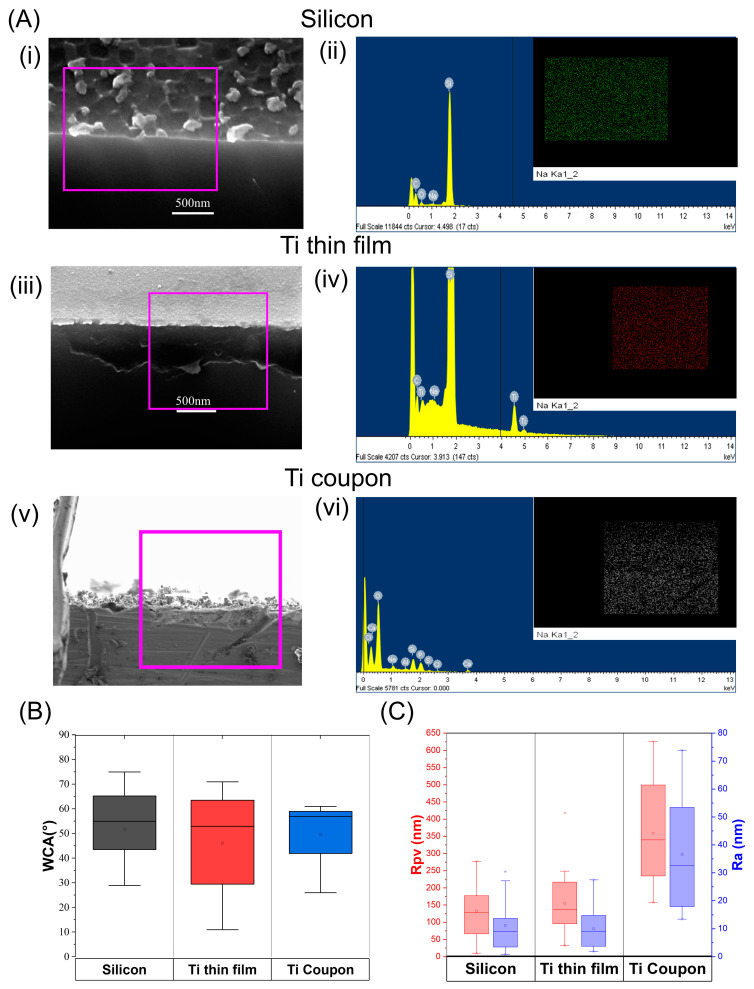
(**A**) Scanning electron microscope (SEM) energy dispersive X-ray (EDX) data of substrates post activation: (**i**) Cross-sectional SEM image of a silicon substrate with area of EDX mapping highlighted in pink, (**ii**) corresponding EDX spectrum and insert of elemental map of sodium, (**iii**) cross-sectional SEM image of a Ti thin-film substrate with area of EDX mapping highlighted in pink, (**iv**) corresponding EDX spectrum and insert of elemental map of sodium, (**v**) cross-sectional SEM image of a Ti coupon substrate with area of EDX mapping highlighted in pink, (**vi**) corresponding EDX spectrum and insert of elemental map of sodium. (**B**) Water contact angle (WCA) in ° measured for each substrate post activation; (**C**) the average roughness (Ra) and peak-to-valley (Rpv) roughness of the three substrates post-activation as measured by atomic force microscopy.

After the activation of the substrates, analysis was performed using SEM-EDX, WCA measurements, and AFM with XEI software. The available literature suggests that when titanium alloy parts are treated with NaOH, it forms a sodium titanate layer, which promotes the formation of apatite [60,61,62,63]. In this work, the SEM-EDX of substrates post NaOH did not show evidence of a sodium titanate layer, likely because the treatment time was less than an hour, i.e., much lower than in other studies. The aim of the activation herein is to generate active sites such as OH-groups on the surface, and so the minimalistic Na amounts are not imperative. Secondly, the presence of a sodium-titanate layer is only possible for bulk titanium parts, and the purpose of our work is to show how a NaOH activation can be used for non-bulk titanium. SEM images and EDX mapping showed that there was no concentrated layer of sodium but low-level sodium detection, <1 at% across the whole sampling area for all substrates, see Figure 1A. Figure 1A(i,ii,v), shows cross-section SEM images of each substrate post activation; samples were cut to perform this, and the pink boxes highlight the EDX area which includes the edge of the substrate. Each pink box has a EDX spectrum Figure 1A(ii,iv,vi) and the insert is a map of the Na content within it. It is shown by the EDX that substrates have a small about of Na detected, but the mapping shows that NA is in no specific pattern; there is no Na layer along the edge. This probably results from Na contamination post NaOH activation. Figure 1A(ii,iv,vi) sub-images show the elemental mapping, where Na was green for silicon (ii), red on Ti thin film (iv), and white on Ti coupon (vi).

Roughness and WCA were also used to investigate the effect of NaOH treatment, with pre-activation data shown in Figures S1–S3. We postulate that the NaOH treatment used rendered the substrates hydrophilic, with all three substrates having WCA < 90°, as seen in Figure 1B. As well as being hydrophilic, all three substrates have similar average WCAs, all measuring between 45 and 55°. Ti Coupons had a WCA distribution of 40–60°, while both novel substrates had a wider distribution. AFM was used to calculate the roughness of each substrate. The Ra of the novel substrates was <30 nm, with the mean Rpv being 125–150 nm. Although this was lower than that of the titanium coupons, the roughness was in the same order of magnitude, as shown in Figure 1C. Thus, these data showed that the sodium content and WCA are similar for all substrates and despite a somewhat difference in roughness, the wettability and chemical functionality of the novel substrates were comparable to the typical titanium part. This is highly important since the deposition process used herein depends on a colloidal solution wetting and bonding with the surface of the substrate, triggering attachment. 

For further clarity, comparative tests were carried out of the Ti thin-film substrate (see Table 1 for the result summary). Samples were activated for different time durations: 5 min (sample 1), 10 min (sample 2), and 15 min (sample 3) before their roughness and WCA was measured. Samples 1, 2, and 3 showed increasing roughnesses with an Rpv of 32.9, 93.2, and 208.9 nm, respectively. The WCA for the samples ranged from 46° to 63°. Samples 1, 2, and 3 were placed in the reaction vessel and underwent HA solution deposition together, thus experiencing matching deposition.

X-ray Photon Spectroscopy (XPS) was performed on the HA-coated substrates to detect small changes in chemical composition at the surface. The resulting chemical compositions calculated from the XPS survey scans (Figures S4–S6) are included in Table 1. Sample 1 which had the lowest roughness had the least mineral attachment, demonstrated by a Ti at% of 14.6. Samples 2 and 3 had no titanium detected; therefore, both had a coherent layer of HA mineral on the surface that was sufficient HA to prevent emission from the sub-layer titanium. Presented in Table 1 are also the results for Ca, P, and O at% from XPS survey scans. Sample 1 has 1.5 at% Ca and no P. Samples 2 and 3 have similar values of 20–22 at% Ca and also 16–18 at% P. 

**Figure 2:**
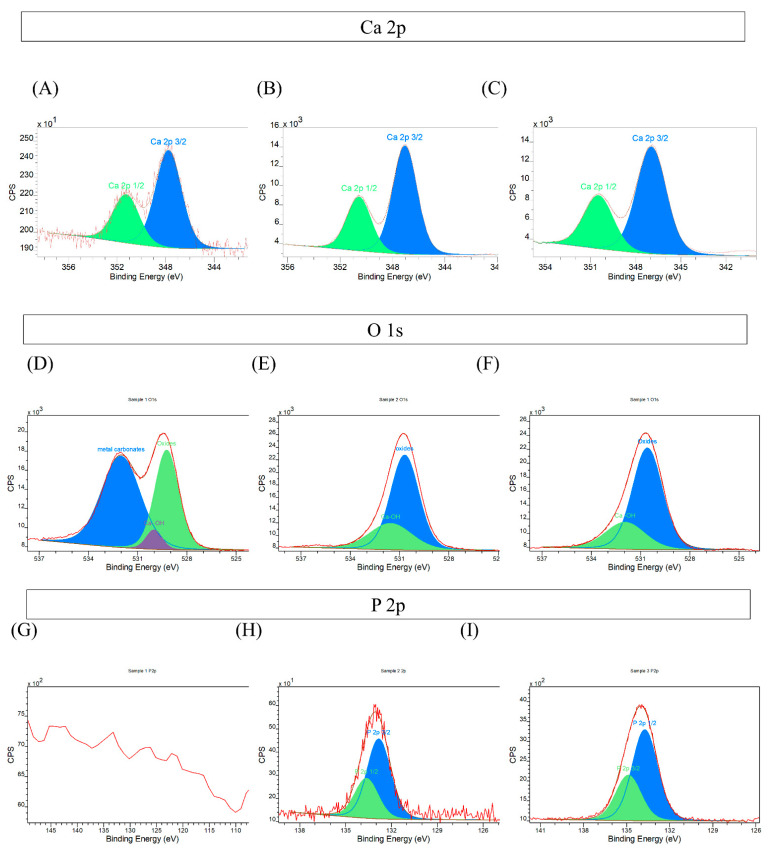
X-ray Photoelectron spectroscopy (XPS) core scans. (**A**) Calcium 2p orbital of Sample 1, (**B**) calcium 2p orbital of Sample 2, and (**C**) calcium 2p orbital of Sample 3. (**D**) Oxygen 1s orbital of Sample 1, (**E**) oxygen 1s orbital of Sample 2, and (**F**) oxygen 1s orbital of Sample 3. (**G**) Phosphorous 2p orbital of Sample 1, (**H**) phosphorous 2p orbital of Sample 2, and (**I**) phosphorous 2p orbital of Sample 3.

A further understanding of the nature of the HA formed on these surfaces was provided through the XPS core scans of O1s, Ca 2p, and P 2p (Figure 2). The core scan of Ca, O, and P reveals the local bond nature of each element and thus provides more information than the XPS Survey scans alone.

1. Calcium: The Ca 2p core scan, Figure 2A, showed that Sample 1 had a low calcium concentration as seen from the CPS and the line shape being noisy. The location of the Ca 2p 3/2 peak at 347.8 eV implies that the low level of Ca is due to contamination or minimal uptake from the colloidal solution. Samples 2 and 3 had significantly higher Ca signals, higher CPS, and smooth lines, Figure 2B,C. The Ca 2p 3/2 peak at the lower binding energies of 347.1 and 346.9 eV, respectively, indicated the deposition of calcium phosphate [64,65].
2. Oxygen: The O 1s core scan, Figure 2D, revealed that Sample 1 had different oxygen bonding compared to Samples 2 and 3, Figure 2E,F, since Sample 1 had a double peak and Samples 2 and 3 had just one peak. All three samples showed a broad O 1s feature at around 532 eV, representative of absorbed hydroxyl species and large oxide features (due to oxides and phosphates) around 530 eV [66]. The double-peak line shape of O 1s for sample 1 indicated that it also had a metal carbonate peak at 533 eV, Figure 2D [67]; this O1s scan for Sample 1 aligns with the XPS survey scan detecting titanium.
3. Phosphorus: Figure 2G is the P 2p core scan of Sample 1, showing no P detection at all through both low CPS and the lack of a peak. Figure 2H is the P 2p core scan of Sample 2 and it shows P detection at a low level. By comparing Sample 2 (Figure 2H) and Sample 3 (Figure 2I), it is clear that Sample 3 had a higher CPS and smoother line shape, implying more P detection. Since Figure 2I showed Sample 3 having the greatest P signal, it was indicative of mineral growth. The binding energies showed that both Sample 2 and 3 had a 2p 3/2 peak at 133 eV, indicative of HA phosphates [64].

We suggest that these three samples are representative of different stages of attachment. Sample 1 was hydrophilic, had the lowest roughness, had minimal calcium, no phosphates, and evidence for metal carbonate deposition. We can conclude that the sample is *activated but limited in sorptive capacity*. Sample 2 was hydrophilic, had greater roughness, and obvious calcium attachment. However, in the O1s scan line shape, there was no evidence of metal carbonates present but only oxide and hydroxide-bonded oxygens, i.e., *calcium started to bond to the active sites of the substrate.* Sample 3 was hydrophilic and had the highest roughness, and had the same calcium and oxygen species and amounts as Sample 2. However, Sample 3 had a much higher P 2p signal, indicating increased phosphate build-up around the previously bonded calcium, i.e., *phosphate groups started to bond to the calcium and HA mineral formation was underway.*

**Figure 3:**
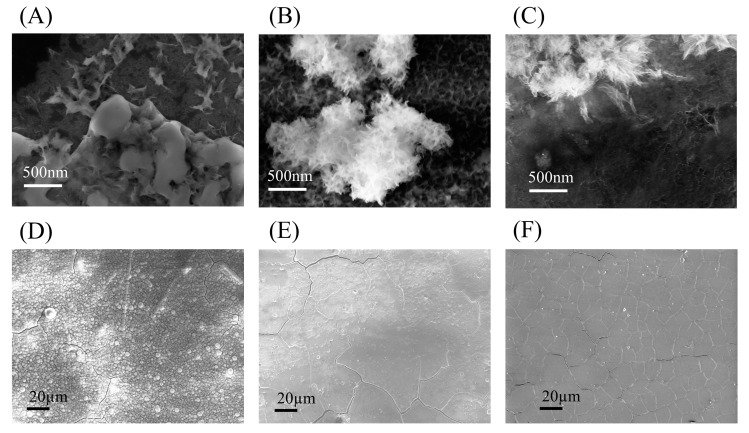
Scanning Electron Microscopy (SEM) images of samples after the first hydroxyapatite deposition run; (**A**) silicon substrate, (**B**) titanium thin film substrate, and (**C**) titanium coupon part. SEM of samples after 6 HA deposition runs; (**D**) silicon substrate; (**E**) Ti thin-film substrate; and (**F**) titanium coupon part.

This XPS study was proof that under the correct conditions of activation, hydrophilicity, and roughness, novel substrates will lend themselves to HA mineral growth under these solution conditions. This mineral growth was further studied for all substrates via SEM imaging after they had undergone one HA deposition run. SEM images showed lighter areas of mineral deposits for all three substrates, as depicted in Figure 3A–C. All three substates generated this initial calcium attachment in discrete areas where there were surface groups for bonding. From this initial nucleation, the heterogeneous growth of a needle-like cobweb structure emerged as phosphate groups bonded to the calcium. Additionally, wider surface coverage was achieved identically for all three substrates after more HA deposition runs, as shown in Figure 3D–F. All samples had good coating integrity and similar morphologies for both Ti samples, but the silicon samples had less repeatable porosity and interconnectivity, as shown in Figure 3D–F. This morphological difference between silicon (Figure 3D) and the titanium substrates (Figure 3E,F) implied a slightly slower and less homogenous growth pattern for silicon. Overall, SEM data further confirm that once a substrate has active sites for bonding, regardless of the substrate, HA mineral growth will progress within this process akin to a bulk titanium part.

**Figure 4:**
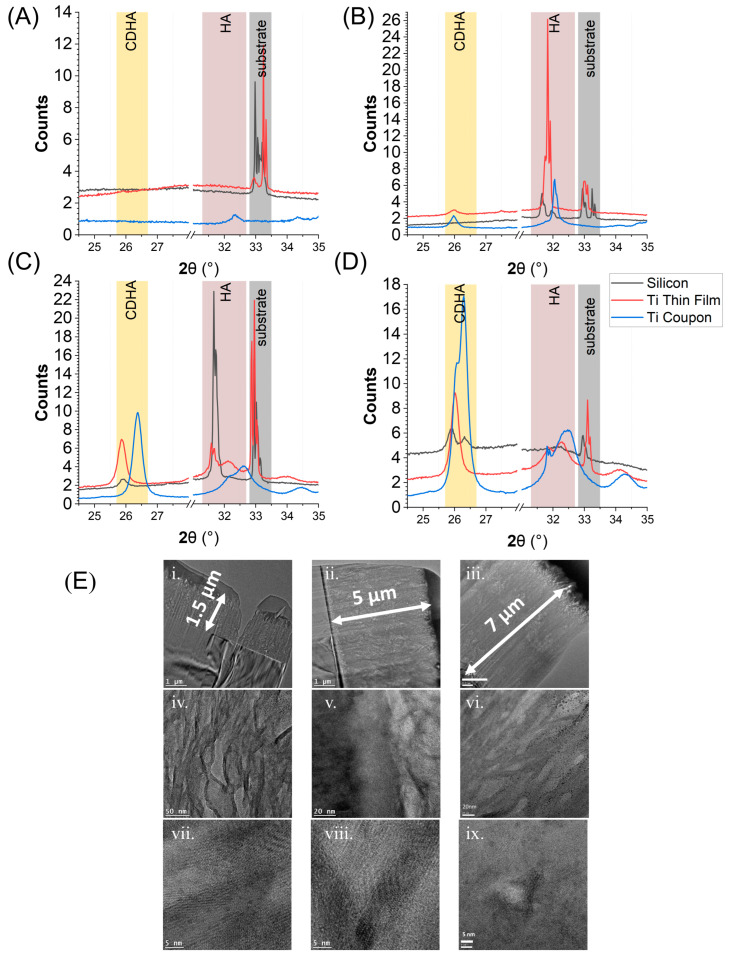
(**A**–**D**): X-ray diffraction (XRD) patterns in terms of counts per second versus diffraction angle of 2θ collected for all three substrates silicon (grey line), Ti thin film (red line), and Ti coupon (blue line). (**A**) Post-activation prior to any HA deposition; (**B**) post 2 HA deposition runs; (**C**) post 4 HA deposition runs; and (**D**) post 6 HA deposition runs. (**E**) Transmission electron microscopy (TEM) cross-sectional images of lamellae cut through HA layers, 1 µm scale, 50 nm scale bar, and higher magnification nanometre scale; (**i**,**iv**,**vi**) cross-section of the HA layer over silicon substrate; (**ii**,**v**,**vii**) cross-section of the HA layer over Ti thin-film substrate; (**iii**,**vi**,**ix**) cross-section of the HA layer over Ti coupon part.

To further probe the growth mechanism through multiple HA deposition runs, XRD patterns were recorded after two, four, and six process runs, as shown in Figure 4B–D. This XRD study compared the evolution of different HA phases as they formed on the different substrates. Both Ti thin films and silicon had substrate peaks around 33°, see Figure 4A. These diminished as the HA layer grew on the surface, Figure 4B. After two process runs, the XRD diffractogram showed that the first detection of mineral growth was characteristic of HA planes (HA), i.e., [211], [112], and [310] around 32°, Figure 4B. However, as the process continued, the mineral formed was calcium-deficient HA (CDHA), as indicated by the [002] plane emerging at 26° [68,69,70], Figure 3C,D. After two process runs, the Ti thin-film parts showed dominant and sharp peaks of HA, but the silicon substrate did not show these until after four process runs, implying slower and less mineral growth for silicon, as shown in Figure 4B,C. The Ti thin-film samples followed the growth pattern of a typical titanium part through the emergence of CDHA and HA characteristic peaks at given stages in the deposition. The XRD study implied that (i) silicon had some CDHA phase, but that it also had the least counts and therefore the least material, and (ii) that the Ti thin film had a lower quantity of the same phases as the Ti coupon.

FIB lamellae of HA films deposited onto all three substrate types were taken, and subsequent TEM cross-sectional analysis allowed for film thickness to be measured. Silicon HA coating measured roughly 1.5 µm, Ti thin films HA coating roughly 5 µm, and Ti coupon parts’ HA coating roughly 6–7 µm in thickness, as shown in Figure 4E(i–iii). TEM imagery showed HA with clear layers from the deposition cycles and pockets of differing crystal orientation within an amorphous matrix, as shown in Figure 4E(iv–vi). High resolution TEM images showed ordered lattice fringes for all three substrates, and, under analysis, these d-spacings were comparable to the XRD data peaks (CDHA of 0.34 nm and Pure HA 0.274–0.28 nm) in Figure 4D [71]. These data again suggest that once HA seeds, it grows similarly for all.

**Figure 5:**
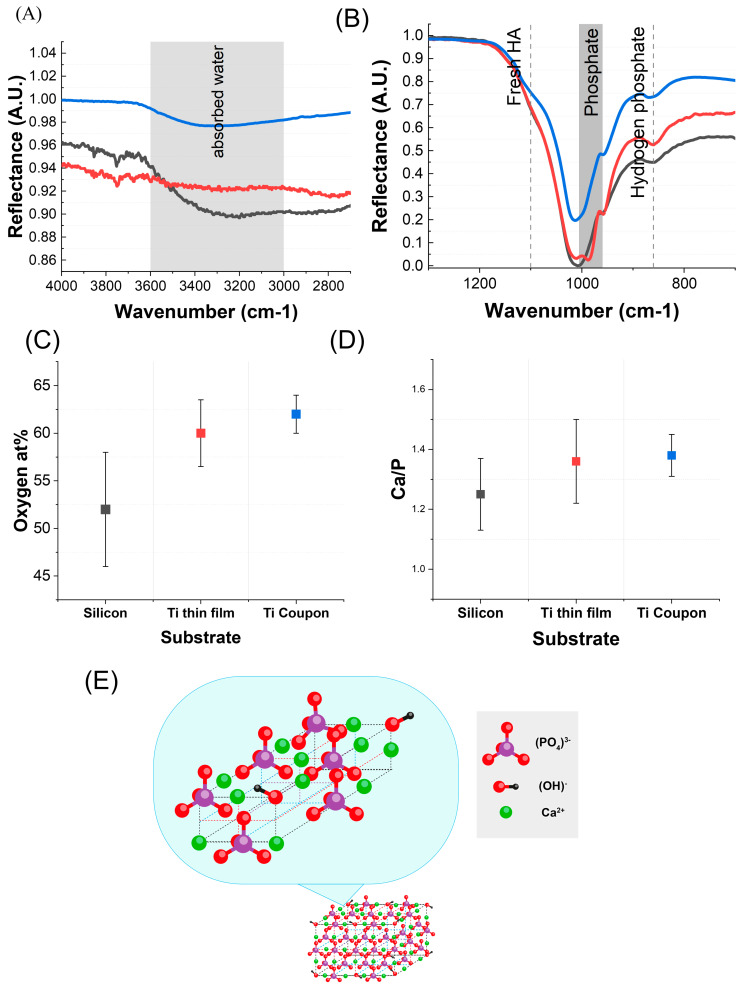
(**A**,**B**) Fourier-transform infrared spectra (FTIR) (**A**) in the absorbed water region of 4000–2000 cm^−1^ and (**B**) in the phosphate region of 1300–700 cm^−1^ of the full deposited HA layer over all three substrates: silicon (grey line), Ti thin film (red line), and Ti coupon (blue line). (**C**,**D**) Collated electron-dispersive X-ray (EDX) data showing mean and error bars for the atomic percentage of (**C**) oxygen (O at%) and (**D**) calcium-to-phosphorus atomic percentage (Ca/P) found in the HA film deposited over silicon, Ti thin film, and Ti coupon parts. (**E**) Crystal structure of hydroxyapatite. For better visualisation, some atoms have been removed from the diagram. The diagram has been constructed from the literature [74,75].

An elemental and chemical analysis of the HA layers was carried out on samples post full deposition using FTIR and SEM-EDX characterisation. From FTIR, the silicon substrate had the strongest indication of absorbed water in the HA layer, as shown in Figure 5A. All substrates had strong vibrational peaks within the known phosphate region of 700 to 1300 cm^−1^, as shown in Figure 5B [72,73]. All substrates demonstrated a matching ratio between the largest peak for *v*^3^ PO_4_^3−^ and shoulder peak of *v*^1^ PO_4_^3−^. Compared to the titanium samples, the silicon samples showed a greater absorbance of HPO_4_^2−^ in agreement with the XRD data having less fully formed HA peaks. Only Ti coupon samples had a shoulder peak for pure HA. 

The EDX analysis of the HA on each substrate supported the calculation of the calcium-to-phosphate ratio (Ca/P) and oxygen atomic percentage (O at %) of the films, as shown in Figure 5C,D. Samples in this study had O at % of 52 ± 6% for silicon, 60 ± 3.5% for Ti thin film, and 62 ± 2% for Ti coupon, Figure 5C. Separately, silicon had the lowest Ca/P at 1.25 ± 0.12, but the Ti thin film was 1.36 ± 0.14, very close to Ti coupon at 1.38 ± 0.07, Figure 5D. Both the relatively low O at % and Ca/P of silicon HA layer illustrate that it is in keeping with the Ti thin-film sample that had early-stage HA growth, i.e., Sample 1 in Table 1.

The unit cell of pure HA, formula Ca_10_(PO_4_)_6_(OH), has hydroxyl ions at the corners of the planes; additionally, phosphate anions and Ca^2+^ cations formed a hexagonal P6_3_/m space group, as seen in Figure 5E. Other phases of HA exist, and from the data herein it became clear that HA films formed from this solution deposition process were a combination of phases. From the literature, pure HA has a Ca/P of 1.67 and O at% of 34.9% and amorphous calcium phosphate (ACP) CaxHy(PO_4_)z∙nH_2_O has a varying Ca/P and O at%. Most pertinent to this work was octacalcium phosphate (OCP) Ca_8_H_2_(PO_4_)_6_·5H_2_O, a phase which has a Ca/P of 1.33 with an O at% of 39.7% [76]. The Ca/P of all three samples heavily indicated the dominant formation of OCP, which supported the XRD observations since the intensity of the OCP peak is greater than the intensity of the HA triple peak. OCP is similar to pure HA but has hydrated layers along the *c* axis of the lattice which would contribute to increased oxygen aided by this aqueous colloidal-solution-based deposition. It is interesting to recall the Ti thin-film samples that underwent XPS analysis, Table 1. These early stages of deposition gave rise to Ca/P in the region of 1.23–1.24 with lower O at% than fully formed samples at EDX. This would demonstrate that the initial phases are calcium phosphate compounds such as Ca_3_(PO_4_)_2_, and we can see from XRD that it is only after two or more deposition runs that HA phases start to form, Figure 4B–D. The rate at which HA phases form is slower for silicon and less-rough Ti thin films than Ti coupons, but they are similar compositionally. Samples in this study all possessed a higher oxygen content than the known phases. This implied the presence of ACP and hydrogen phosphates, but also the presence of adsorbed water and oxides around the lattice. Silicon samples showed the highest % of O, again implying the lowest pure HA content.

## 4. Conclusions

A successful method of deposition of hydroxyapatite films using a novel colloidal solution deposition process was developed and its efficacy in coating non-bulk titanium parts was confirmed in this work. SEM-EDX data of all substrates post NaOH activation show that there is equivalent low-level sodium present across the surface of all three. However, the importance of the formation of a sodium titanate film in an activation process is very much less than previously thought. AFM and WCA analysis showed that although the two novel-type substrates were less rough than a typical titanium part, they had similar hydrophilicity. XPS data gathered from HA on Ti thin-film samples proved that the roughness of a novel substrate will alter the propensity of the substrate for HA deposition showing that, once suitable activation is reached, HA will grow. SEM data suggested that HA mineral deposits were generated after the first process run in discrete regions of the surface. SEM also showed that after six process runs, all three substates had widespread HA film coverage. XRD revealed the pathway by which HA grows within this process for a typical titanium part and that Ti thin-film parts follow this mechanism closely, but also that silicon has a slower growth mechanism. Through TEM analysis, the thickness and crystallinity of layers were compared. While silicon was found to have less material, all three substrates had crystalline regions within an amorphous matrix. The chemical composition of the film, determined from FTIR and SEM-EDX data, showed that all three had ACP and OCP present with a limited proportion of pure HA, but that the silicon had higher impurities.

Overall, we confirmed that a Ti thin film can support the growth of an HA layer in a similar manner as a Ti coupon part. This opens the possibilities of coating non-bulk metallic orthopaedic implants, such as plastic, via simple titanium deposition. Ongoing work suggests these methods can be applied for many different substrates.

## Figures and Tables

**Table 1:** Tabulated data of the water contact angle and roughness of Ti thin-film samples post-activation, and their resulting chemical composition as measured by XPS post HA deposition.

Ti Thin-Film Sample	WCA [°]	Rpv [nm]	Ra [nm]	Ca at%	P at%	O at%	Ti at%
1	46	32.9	2.4	1.5	0	47.9	14.6
2	63	93.2	10.8	21.9	17.8	45.9	0
3	58	205.9	33.7	20.8	16.7	42.7	0

## Data Availability

Data are contained within the article or Supplementary Material.

## Supplementary Materials

The following supporting information can be downloaded at https://www.mdpi.com/article/10.3390/nano13172483/s1. Figure S1: WCA in measured for each substrate pre and post activation. Figure S2: The peak-to-valley (Rpv) roughness of the three substrates pre- and post-activation as measured by atomic force microscopy. Figure S3: The average roughness (Ra)of the three substrates pre- and post-activation as measured by atomic force microscopy. Figure S4: X-Ray Photoelectron spectroscopy analysis, showing the Survey Scan of HA coated titanium thin film (Sample 1), Y-axis: counts per second [CPS] versus X-axis: binding energy [eV]. Figure S5: X-Ray Photoelectron spectroscopy analysis, showing the Survey Scan of HA coated titanium thin film (Sample 2), Y-axis: counts per second [CPS] versus X-axis: binding energy [eV]. Figure S6: X-Ray Photoelectron spectroscopy analysis, showing the Survey Scan of HA coated titanium thin film (Sample 3), Y-axis: counts per second [CPS] versus X-axis: binding energy [eV].
