# Supplementary material for: Characterising Hydroxyapatite Deposited from Solution onto Novel Substrates: Growth Mechanism and Physical Properties
Source: Nanomaterials (Basel). 2023 Sep 3;13(17):2483. doi: 10.3390/nano13172483 (PMC10489777; doi:10.3390/nano13172483)
Supplement: Supplementary file 1 [file nanomaterials-13-02483-s001.zip › nanomaterials-2581224-supplementary.pdf]

Supplementary Materials

Characterising Hydroxyapatite Deposited from Solution onto Novel Substrates: Growth Mechanism and Physical Properties

Bríd Murphy [1,2,*], Jhonattan Baez [1,2] and Mick A. Morris [1,2,*]

[1] Advanced Materials & Bioengineering Research Centre (AMBER), Trinity College Dublin, Dublin 2, D02 CP49 Dublin, Ireland

[2] School of Chemistry, Trinity College Dublin, Dublin 2, D02 PN40 Dublin, Ireland

* Correspondence: murphb52@tcd.ie (B.M.); morrism2@tcd.ie (M.A.M.)

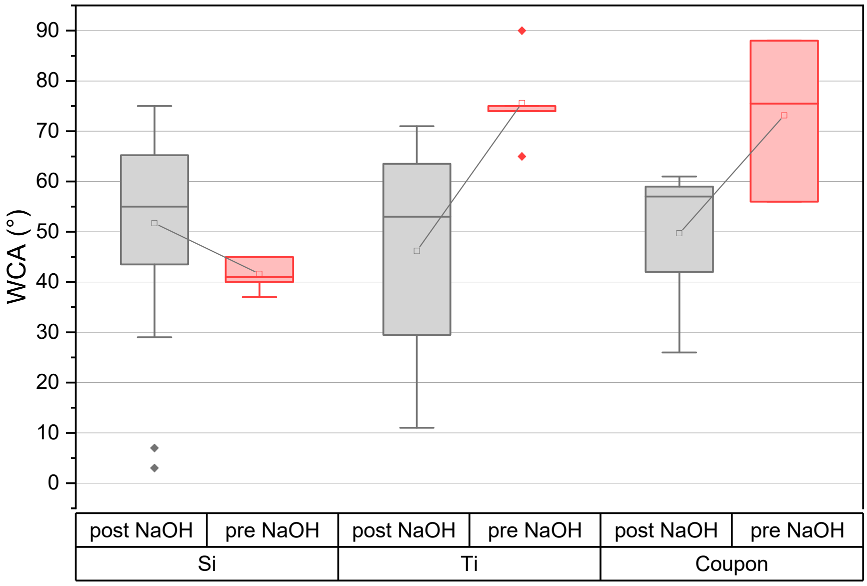

Figure 1. WCA in ° measured for each substrate pre and post activation.

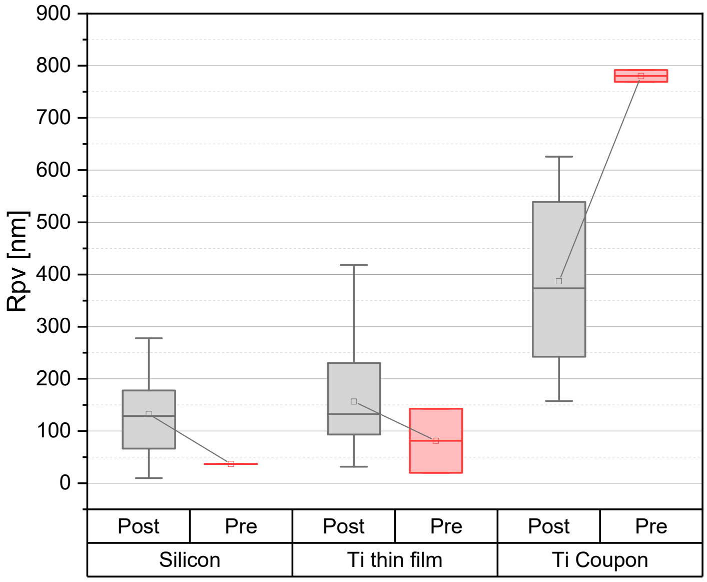

Figure 2. The peak-to-valley (Rpv) roughness of the three substrates pre- and post-activation as measured by atomic force microscopy.

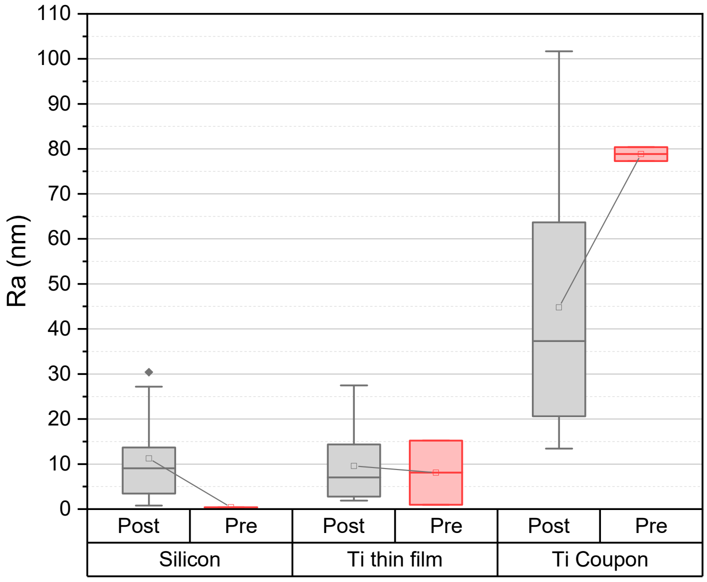

Figure 3. The average roughness (Ra)of the three substrates pre- and post-activation as measured by atomic force microscopy.

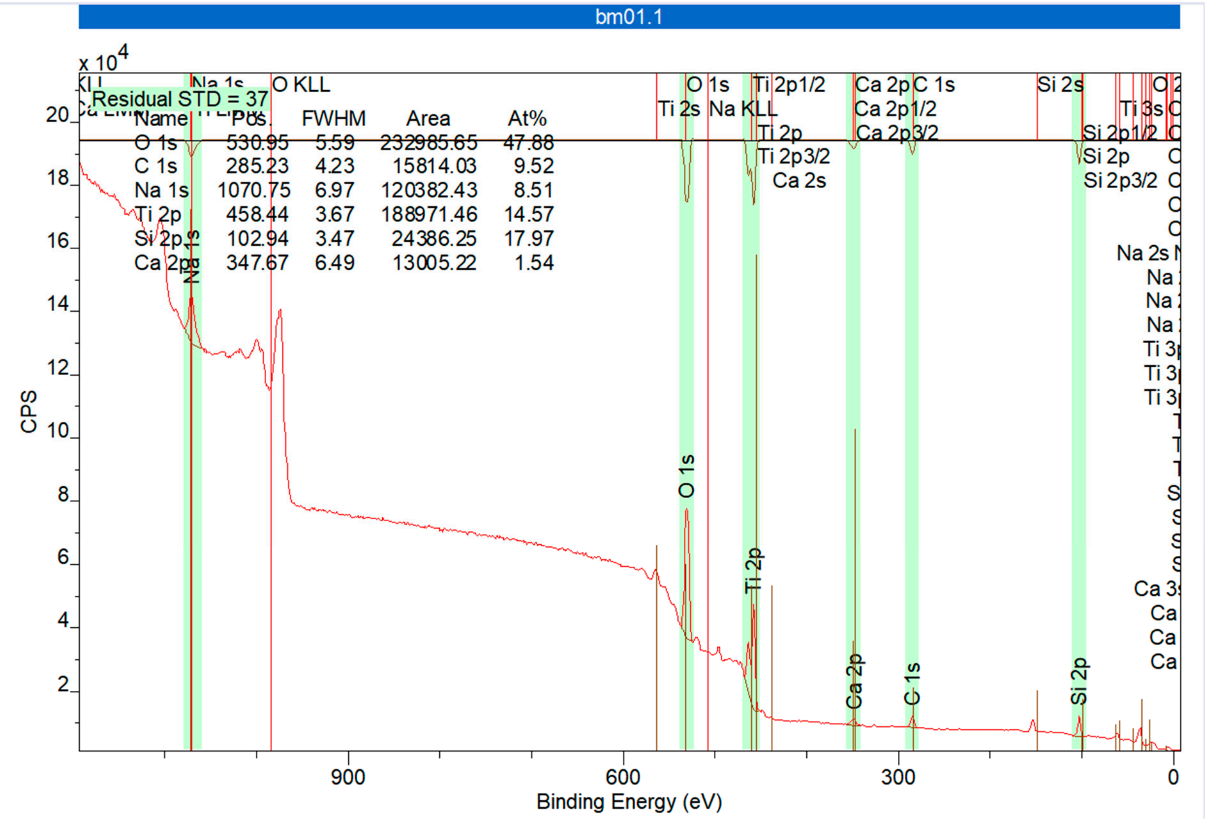

Figure 4. X-Ray Photoelectron spectroscopy analysis, showing the Survey Scan of HA coated titanium thin film (Sample 1), Y-axis: counts per second [CPS] versus X-axis: binding energy [eV].

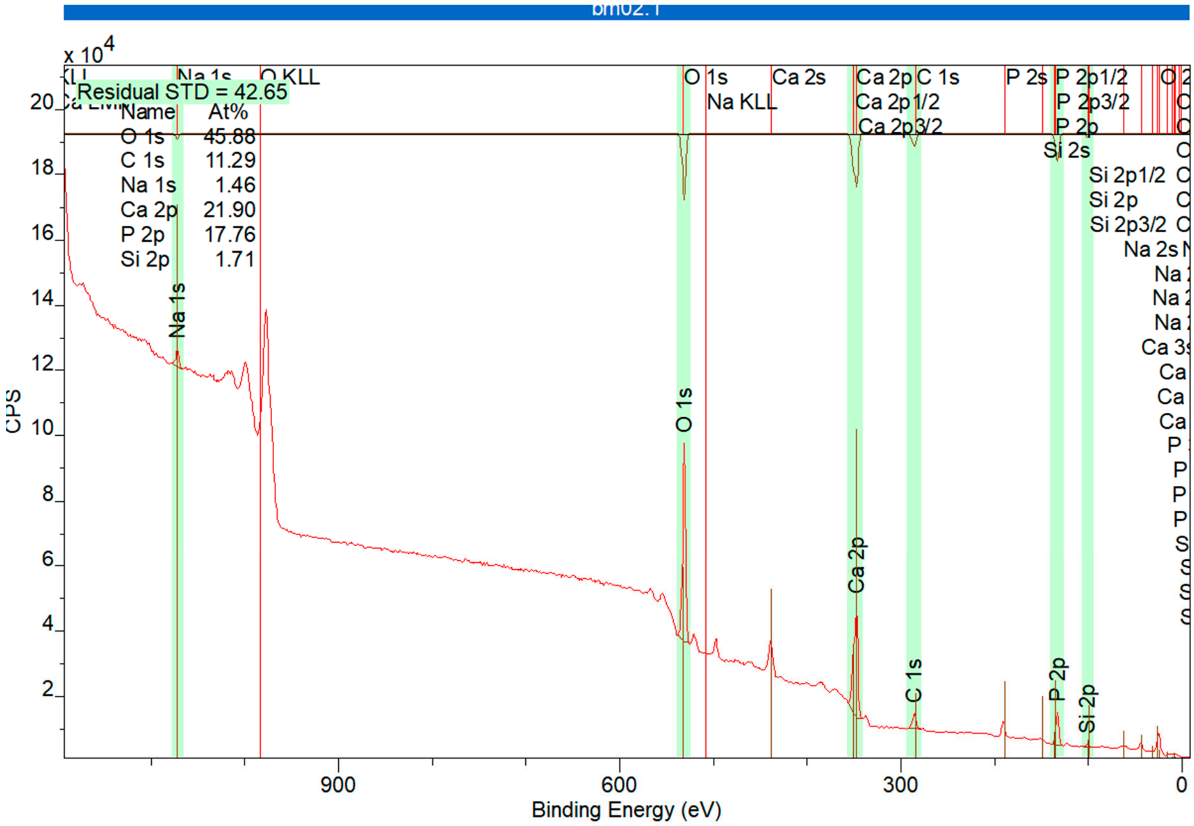

Figure 5. X-Ray Photoelectron spectroscopy analysis, showing the Survey Scan of HA coated titanium thin film (Sample 2), Y-axis: counts per second [CPS] versus X-axis: binding energy [eV].

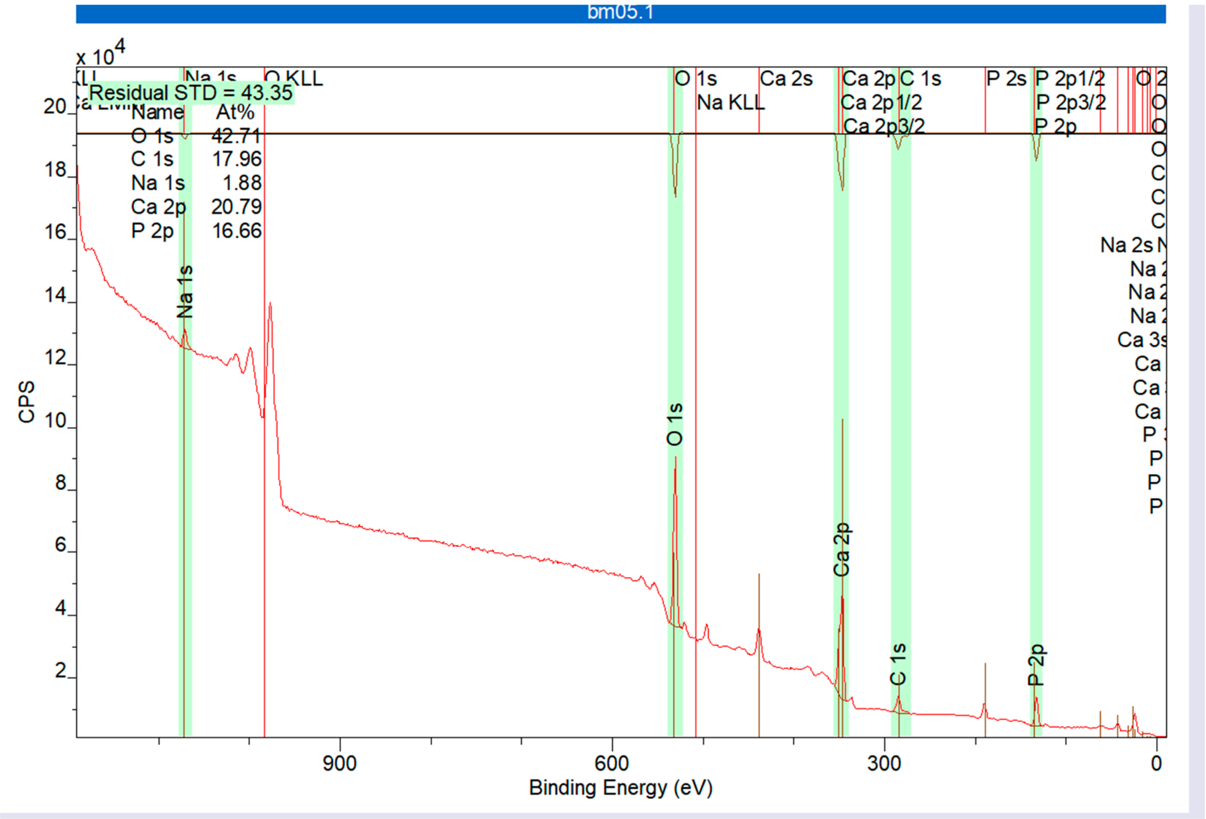

Figure 6. X-Ray Photoelectron spectroscopy analysis, showing the Survey Scan of HA coated titanium thin film (Sample 3), Y-axis: counts per second [CPS] versus X-axis: binding energy [eV].
